# Automatic construction of statistical shape models using deformable simplex meshes with vector field convolution energy

**DOI:** 10.1186/s12938-017-0340-0

**Published:** 2017-04-24

**Authors:** Jinke Wang, Changfa Shi

**Affiliations:** 1 Department of Software Engineering, Harbin University of Science and Technology, Rongcheng, 264300 China; 2 Mobile E-business Collaborative Innovation Center of Hunan Province, Hunan University of Commerce, Changsha, 410205 China

**Keywords:** Shape model construction, Shape correspondence establishment, Deformable models, Simplex meshes, VFC energy, Greedy algorithm

## Abstract

**Background:**

In the active shape model framework, principal component analysis (PCA) based statistical shape models (SSMs) are widely employed to incorporate high-level a priori shape knowledge of the structure to be segmented to achieve robustness. A crucial component of building SSMs is to establish shape correspondence between all training shapes, which is a very challenging task, especially in three dimensions.

**Methods:**

We propose a novel mesh-to-volume registration based shape correspondence establishment method to improve the accuracy and reduce the computational cost. Specifically, we present a greedy algorithm based deformable simplex mesh that uses vector field convolution as the external energy. Furthermore, we develop an automatic shape initialization method by using a Gaussian mixture model based registration algorithm, to derive an initial shape that has high overlap with the object of interest, such that the deformable models can then evolve more locally. We apply the proposed deformable surface model to the application of femur statistical shape model construction to illustrate its accuracy and efficiency.

**Results:**

Extensive experiments on ten femur CT scans show that the quality of the constructed femur shape models via the proposed method is much better than that of the classical spherical harmonics (SPHARM) method. Moreover, the proposed method achieves much higher computational efficiency than the SPHARM method.

**Conclusions:**

The experimental results suggest that our method can be employed for effective statistical shape model construction.

## Introduction

Medical image segmentation is a prerequisite for various clinical applications, such as medical diagnosis, treatment planning and image-guided surgery. In the last few decades, numerous medical image segmentation methods have been proposed [1]. Among all these proposed methods, active shape model (ASM) method [2] has achieved state-of-the-art segmentation accuracy [3]. ASM uses principal component analysis (PCA) [4] based statistical shape models (SSMs) to incorporate high-level a priori shape knowledge of the structure to be segmented to achieve robustness. SSMs can capture the range of shape variability for the structure to be segmented based on a set of training samples. A crucial component of building SSMs is to establish shape correspondence between all training shapes, which is a very challenging task, especially in three dimensions. Also, this component to a great extent determines the final performance of the learned shape prior models. Various methods for establishing shape correspondence have been proposed in the literature, and these methods can be roughly divided into five major categories [3]: mesh-to-mesh registration [5, 6], mesh-to-volume registration [11], volume-to-volume registration [7], parameterization-to-parameterization registration [8], and population-based optimization [9]. Alternatively, these methods can be classified as pairwise correspondence methods [10] and groupwise methods [11]. In particular, Gollmer et al. [12] presented a thorough evaluation of different groupwise correspondence methods, including their objective functions, re-parameterization schemes, and optimization strategies. However, a major limitation of current shape correspondence establishment methods, such as the spherical harmonics (SPHARM) method [8], is that they are very computationally intensive and the structure to be modeled has to be of the same topology as the sphere (i.e., of zero genus).

Deformable models [13] have been quite popular in medical image analysis, particularly in image segmentation [14]. Deformable models are contours or meshes that evolve under the constraints of both internal and external energy to segment the object of interest. In this paper, to overcome the above-mentioned limitations of current shape correspondence establishment methods, we propose an automatic deformable surface model based shape correspondence establishment method to improve the accuracy and reduce the computational cost. Specifically, we develop a greedy algorithm based deformable simplex mesh that uses vector field convolution (VFC) as the external energy. Simplex meshes [15] are efficient and versatile surface representation for physics-based deformable models. These models are formulated based on the concept of force equilibrium, which includes internal and external forces, rather than the original energy minimization framework [13]. The greedy algorithm that minimizes a variational energy functional was introduced in [16] as an alternative to the physics-based method for deformable model evolution. It has been shown that the greedy algorithm outperformed physics-based method both in computation cost and segmentation accuracy [17]. Therefore, we adapt the greedy algorithm to perform the evolution of the deformable simplex meshes. VFC field [18] is a widely used static external force for physics-based deformable models that can guide the active contour into long and thin boundary. Furthermore, in comparison with the classical gradient vector flow (GVF) external force [19–21], VFC force shows superior robustness to noise and initialization, much less computational cost (3–10 times less), and changing flexibility [18]. Also, GVF force in homogeneous regions of the image has demonstrated to be a special case of VFC force under certain condition [18]. In order to overcome the main issues of traditional external energy (i.e., sensitivity to shape initialization and poor convergence to the long and thin boundary concavities), we adapt the VFC field for greedy algorithm as external energy. Therefore, our proposed method integrates the computational simplicity of simplex meshes, speed of the greedy algorithm, and robustness of VFC in a unified system.

The deformable models are known to be sensitive to shape initialization [19]. In order to get accurate and robust results, we develop an automatic shape initialization method to derive an initial shape that has high overlap with the object of interest, such that the deformable models can then evolve more locally. We apply our proposed deformable surface model to the application of femur statistical shape model construction to illustrate its accuracy and efficiency. This paper significantly extends our preliminary conference paper on GVF-based deformable simplex meshes [21] by (1) introducing a new VFC external energy, (2) developing an automatic shape initialization method, and (3) applying our method to establish shape correspondence and construct statistical shape models.

## Related work

In this section, we briefly review the related mesh-to-volume registration based methods for establishing shape correspondence.

In mesh-to-volume registration based methods, a landmarked deformable surface model is fitted to all the segmented training images. After the evolution of deformable model has converged, the final landmark locations of the deformable template determine the point correspondence [3]. Kaus et al. [22] presented a 3-D elastically deformable model for the automated establishment of shape correspondence from segmented images, where a triangulated deformable template was adopted to segment binary volumetric images of the remaining training samples. Their method can approximate and predict unseen shapes well. Shang and Dossel [20] then extended the above-mentioned method by adopting the force equilibrium concept of deformable surface model, which included the internal forces and the external GVF image forces [19]. Compared with traditional simple gradient image forces, their use of GVF forces greatly improved the accuracy of shape correspondence. Shen et al. [23] proposed an adaptive-focus deformable model (AFDM) to establish shape correspondence based on the hand-labeled 3-D brain images. They introduced an attribute vector for each landmark of the deformable template to preserve its geometric shape while evolution. However, there exit failure cases where the deformable template has to be manually pulled towards the boundary. Later, Zhao and Teoh [24] extended the AFDM method by developing a “bridge over” framework and used it to construct SSMs of brain ventricles. Compared with the original AFDM method, their new method achieved more accurate shape representation, but at the cost of more computation time. Clogenson et al. [25] proposed a model-fitting based approach to establish shape correspondence from manually segmented CT scans. An initial Procrustes alignment between the training data and the reference surface mesh is performed, followed by a non-rigid registration between the reference surface mesh and distance maps of the training label maps. The non-rigid registration is formulated as a model-fitting problem, where a Gaussian Process prior is employed to model smooth deformations of the reference surface mesh.

## Background

In this section, we briefly show the main geometry of simplex meshes [21], the reader is referred to [15] for the detailed definition. These geometry information, especially the metric parameters and simplex angle, will be used to define the internal energy of our proposed deformable simplex meshes.

**Fig. 1:**
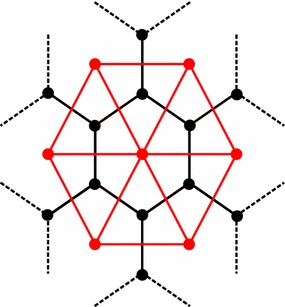
Diagram showing the duality between 2-simplex meshes (*black lines*) and triangular meshes (*red lines*)

A *k*-simplex mesh is a polygonal mesh where each vertex has precisely $(k+1)$ neighbors. In this study, we will only consider 2-simplex meshes defined in $\mathbb {R}^3$, which are also the dual of the triangular meshes (see Fig. 1). Each vertex $P_i$ is linked to exactly 3 distinct vertices $(P_{N_1(i)},P_{N_2(i)},P_{N_3(i)})$, which form the tangent plane at $P_i$ with its normal vector $\mathbf {n}_i$. Local geometry around a vertex $P_i$ involves the circumscribed circle $S_1$ with center $C_i$ and radius $r_i$ of the triangle $(P_{N_1(i)},P_{N_2(i)},P_{N_3(i)})$, and the circumscribed sphere $S_2$ with center $O_i$ and radius $R_i$ of the tetrahedron $(P_i,P_{N_1(i)},P_{N_2(i)},P_{N_3(i)})$ (please refer to Fig. 8 of [15]).

The metric parameters $(\epsilon _{1i},\epsilon _{2i},\epsilon _{3i})$ at a vertex $P_i$ are the barycentric coordinates of the orthogonal projection $F_i$ of $P_i$ onto the tangent plane with respect to the triangle $(P_{N_1(i)},P_{N_2(i)},P_{N_3(i)})$:

1 $$F_i = \epsilon _{1i}P_{N_1(i)}+\epsilon _{2i}P_{N_2(i)}+\epsilon _{3i}P_{N_3(i)},$$

2 $$\epsilon _{1i}+\epsilon _{2i}+\epsilon _{3i}=1.$$

The simplex angle $\phi _i \in {[-\pi , \pi ]}$ at $P_i$ is defined as:

3 $$\cos {(\phi _i)}= \frac{ \left\| C_i-O_i\right\| }{ R_i } \text { sign} \left( (C_i-O_i) \cdot \mathbf {n}_i \right) .$$

The height $L(r_i,d_i,\phi _i)$ of $P_i$ with respect to the tangent plane is defined as:

4 $$L(r_i,d_i,\phi _i) = \frac{ (r^2-d^2)\tan (\phi _i) }{ \epsilon \sqrt{ (r^2-d^2) \tan ^2(\phi _i) }+r_i },$$

with

$$\epsilon = {\left\{ \begin{array}{ll} 1, &\quad\text {if }|\phi _i| < \pi /2 \\ -1, &\quad\text {if }|\phi _i| > \pi /2, \end{array}\right. }$$

where $d_i=\left\| F_i-C_i\right\|$. Therefore, the position of $P_i$ can be uniquely defined in terms of its three neighbors:

5 $$P_i = \epsilon _{1i}P_{N_1(i)}+\epsilon _{2i}P_{N_2(i)}+\epsilon _{3i}P_{N_3(i)}+ L(r_i,d_i,\phi _i) \mathbf {n}_i.$$

While the metric parameters control the position of the orthogonal projection $F_i$ of $P_i$ onto the tangent plane, the local mean curvature $H_i = \frac{ \sin {(\phi _i)} }{ r_i }$ at $P_i$ is controlled by the simplex angle.

## Methods

**Fig. 2:**
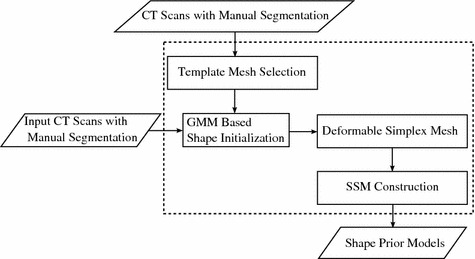
Flowchart of the proposed method for statistical shape model construction

In this section, we describe the details of the proposed deformable surface model based statistical shape models construction method. It adopts a greedy algorithm which allows simplex meshes to converge on the object of interest under the constraints of both internal energy and VFC external energy. The whole procedure is illustrated in Fig. 2.

### Automatic initialization of deformable surface models

In order to get accurate and robust results, we develop an automatic shape initialization method to derive an initial shape that has high overlap with the object of interest, such that the deformable models can then evolve more locally. Given the training samples with ground truth segmentation, we firstly obtain their simplex mesh representations, which are defined as the dual of the triangular mesh derived via Marching Cubes algorithm [26] and mesh smoothing methods [27]. To minimize bias towards the chosen initial shape and make the deformable simplex meshes more robust to local minima, we then choose the training sample similar to an average femur shape as the template mesh for the deformable models.

In order to obtain a good initial shape for the deformable models, we employ the Gaussian mixture model (GMM) based point set registration method [28] by aligning the template mesh with other training meshes via an affine transformation. In GMM based registration method, the main idea is to represent the point sets to be registered as Gaussian mixture models, and then align the two corresponding Gaussian mixtures by minimizing their $L_2$ distance. Specifically, we represent the point sets of the template mesh as a Gaussian mixture $f(\mathbf {x}) = \sum _{ i=1 }^{m} {\alpha _{i} \phi (\mathbf {x} | \mu _{i},\Sigma _{i})}$, where $\alpha _{i}$ is the weight of each component, $\phi (\mathbf {x} | \mu _{i},\Sigma _{i})$ is a Gaussian distribution with mean $\mu _{i}$ and covariance variance $\Sigma _{i}$. Similarly, the point sets of other training meshes are represented by $g(\mathbf {x}) = \sum _{ j=1 }^{n} {\beta _{j} \phi (\mathbf {x} | \nu _{j},\Gamma _{i})}$. An affine transformation can be represented by a $3 \times 3$ matrix $\mathbf {A}$, and a translation vector $\mathbf {t}$. For convenience, the matrix $\mathbf {A}$ is factorized as an orthogonal matrix $\mathbf {Q}$ and a symmetric positive definite matrix $\mathbf {S}$, i.e., $\mathbf {A}=\mathbf {Q}\mathbf {S}$ [28]. Then the affine transformation (i.e., $\mathbf {A}$ and $\mathbf {t}$) can be found by minimizing the following $L_2$ distance between Gaussian mixtures $f(\mathbf {x})$ and $g(\mathbf {x})$ [28]:

6 $$d_{L_2}(f,g,\mathbf {A},\mathbf {t}) = \int {(g-f_{\mathbf {A},\mathbf {t}})^2dx} = \int {(f_{\mathbf {A},\mathbf {t}}^2-2f_{\mathbf {A},\mathbf {t}}g+g^2)dx},$$

where $f_{\mathbf {A},\mathbf {t}}(\mathbf {x})= \sum _{ i=1 }^{m} {\alpha _{i} \phi (\mathbf {x} | \mathbf {A}\mu _{i}+\mathbf {t},\mathbf {Q}(\Sigma _{i})\mathbf {Q}^T)}$ is the transformed distribution of the template mesh. Since $\int {g^2}$ is independent of the affine transformation, we only need to consider $\int {f_{\mathbf {A},\mathbf {t}}^2}$ and $\int {f_{\mathbf {A},\mathbf {t}}g}$. Fortunately, both terms has closed-form expressions, for the latter term:

7 $$\int {f_{\mathbf {A},\mathbf {t}}g \, dx} = \sum _{ i=1 }^{m} {\sum _{ j=1 }^{n} {\alpha _{i}\beta _{j} \phi (\mathbf {x} | \mu _{i}-\nu _{j},\mathbf {Q}(\Sigma _{i})\mathbf {Q}^T+\Gamma _{i})}}.$$

Considering that the gradients associated with affine transformation have analytical solutions [28], fast gradient-based numerical optimization techniques (e.g., Quasi-Newton methods) can be deployed to minimize the objective function.

After deriving the affine transformation (i.e., $\mathbf {A}$ and $\mathbf {t}$), we used it to transform the template mesh to the space of other training meshes, resulting in the initial shape for the deformable models.

### Evolution method

**Fig. 3:**
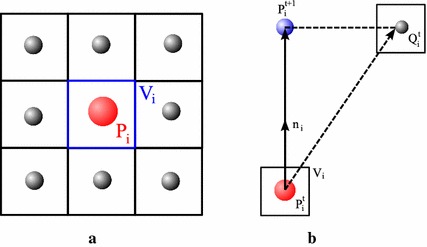
The greedy algorithm: **a** the energy function is calculated at vertex $P_i$ and voxels in the $w\times w\times w$ cubic window around $V_i$, and the point with the smallest energy is selected as the target position of $P_i$. **b** The vertex $P_i$ is moved only along its normal direction $\mathbf {n}_i$. For illustration purpose, only a 2-D window is showed

We adopt the greedy algorithm [16] as evolution method to guide deformable 2-simplex meshes to the object of interest (e.g., edges) [21]. Let $V_i$ be the voxel in the volumetric image containing vertex $P_i$. During each iteration, a $w\times w\times w$ cubic window around $V_i$ is searched, and the energy is computed at each voxel within the window (see Fig. 3a). The energy at vertex $P_i$ is defined as a combination of both internal and external energy normalized within the window:

8 $$\begin{aligned} {\varvec{E}(P_i) }&= {\alpha \varvec{E}_{int}(P_i) + \beta \varvec{E}_{ext}(P_i)} \\&= {\alpha \left( \varvec{E}_{Tangent}(P_i) + \varvec{E}_{Normal}(P_i) \right) + \beta \varvec{E}_{VFC}(P_i)}, \end{aligned}$$

where $\varvec{E}_{Tangent}$ and $\varvec{E}_{Normal}$ are internal energy, $\varvec{E}_{VFC}$ is the VFC external energy, and $\alpha > 0$, $\beta > 0$ are weighting parameters that control the relative influence of internal and external energy. The internal energy regularizes the rigidity and elasticity of the deformable models, while VFC external energy attracts the deformable models to the object of interest (e.g., edges).

We define ${N_i}^{t}$ as the $w\times w\times w$ cubic window around voxel $V_i$. The position ${Q_i}^{t}$ with minimum energy within the window ${N_i}^{t}$ is chosen as:

9 $${Q_i}^{t}={\mathop{\text{arg min}}\limits_{{P_j}\in {N_i}^{t}}} \,\varvec{E}(P_j).$$

In order to guarantee a stable and smooth deformation of the 2-simplex meshes, the vertex $P_i$ is moved only along its normal direction $\mathbf {n}_i$, rather than directly moved to ${Q_i}^{t}$ as in the classical greedy algorithm (see Fig. 3b):

10 $${P_i}^{t+1} = {P_i}^{t}+ \left( ({Q_i}^{t}-{P_i}^{t})\cdot \mathbf {n}_i \right) \mathbf {n}_i.$$

The overall pseudocode of the greedy algorithm is summarized in Algorithm 1 [21].
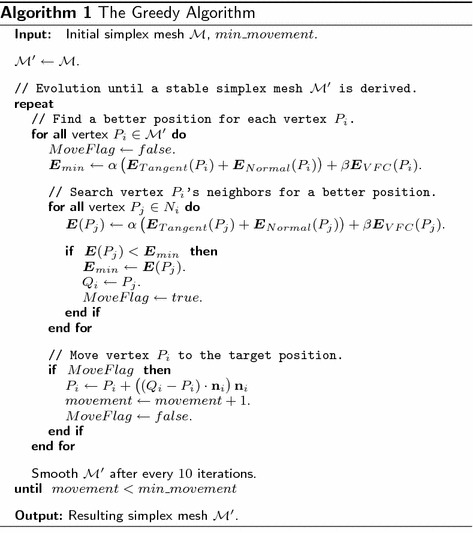


### Internal energy computation

The internal energy maintains the geometrical regularity of the deformable 2-simplex meshes. We adopt the original internal force proposed in [15] for greedy algorithm as internal energy, which is decomposed into tangential energy and normal energy [21]:

11 $$\begin{aligned} {\varvec{E}_{int}(P_i) }&= {\varvec{E}_{Tangent}(P_i) + \varvec{E}_{Normal}(P_i)} \\&= {{\left\| \frac{1}{3}( P_{N_1(i)}+P_{N_2(i)}+P_{N_3(i)} )-F_i \right\| }^2 + \;{\left\| L(r_i,d_i,\tilde{\phi }_i)-L(r_i,d_i,\phi _i) \right\| }^2,} \end{aligned}$$

where $\tilde{\phi }_i$ is the reference simplex angle defined as the mean of neighboring vertices’ simplex angles. The tangential energy measures the distance of the orthogonal projection $F_i$ of $P_i$ from the center of gravity of its three neighbors in the tangent plane. When the energy achieves the minimum, it ensures that the vertices exhibit uniformly on the surface. While the normal energy keeps a local smoothing simplex mesh through a $C^2$ smoothness constraint.

### VFC external energy computation

Vector field convolution (VFC) field [18] is a widely used static external force for physics-based deformable models. It largely solves the problems associated with traditional external force and can guide the active contour into long and thin boundary. Furthermore, in comparison with the classical GVF external force [19–21], VFC force shows superior robustness to noise and initialization, and much less computational cost [18]. The three-dimensional VFC field $\mathbf {v}({x, y, z})=[u({x, y, z}), v({x, y, z}), w({x, y, z})]$ is defined as the convolution of a vector field kernel $\mathbf {k}({x, y, z})$ with the edge map *f*(*x*, *y*, *z*) generated from the input image *I*(*x*, *y*, *z*):

12 $$\mathbf {v}({x, y, z}) = f({x, y, z}) \otimes \mathbf {k}({x, y, z}),$$

where $\otimes$ denotes linear convolution. The basic principle behind this formula is to propagate the gradient vectors of input image *I*(*x*, *y*, *z*) into homogeneous image regions via image convolution, such that the deformable models have more chances to move towards the object of interest (e.g., edges).

The edge map *f*(*x*, *y*, *z*) for gray-level input images is usually defined as the gradient magnitude of the blurred image:

13 $$f({x, y, z}) = | \nabla [G_{\sigma }({x, y, z})] \otimes I({x, y, z})|,$$

where $G_{\sigma }({x, y, z})$ is a 3-D Gaussian function with standard deviation $\sigma$, and $\nabla$ is the gradient operator. The vector field kernel $\mathbf {k}({x, y, z})=[u_k({x, y, z}), v_k({x, y, z}), w_k({x, y, z})]$ can be calculated as follows:

14 $$\mathbf {k}({x, y, z}) = m({x, y, z}) \mathbf {n}({x, y, z}),$$

where *m*(*x*, *y*, *z*) is the magnitude of the vector at (*x*, *y*, *z*) and $\mathbf {n}({x, y, z})$ is the unit vector from (*x*, *y*, *z*) to the kernel origin (0, 0, 0) given as:

$$\mathbf{{n}}(x,y,z) = \left\{ {\begin{array}{lll} {[0,0,0],}&\quad{\mathrm{{if }}(x,y,z) = (0,0,0),}\\ {[ - \frac{x}{r}, - \frac{y}{r}, - \frac{z}{r}],}&\quad{\mathrm{{otherwise}},} \end{array}} \right.$$

where $r=\sqrt{x^2 + y^2 + z^2}$ is the distance from the kernel origin (0, 0, 0).

The magnitude of the vector field kernel *m*(*x*, *y*, *z*) plays a major role in generating the VFC field. Considering that the influence of the object of interest should be diminished as the deformable models become further away, then two types of magnitude functions can be defined as decreasing functions of the distance from the kernel origin [18]:

15 $$m_1({x, y, z})=( r + \varepsilon )^{-\gamma },$$

16 $$m_2({x, y, z})= {\rm exp} \left( \frac{-r^2}{\zeta ^2}\right),$$

where $\gamma > 0$ and $\zeta > 0$ are parameters that control the decrease of the magnitude, and $\varepsilon > 0$ is a small constant for the prevention of division by zero at the kernel origin. The parameters $\gamma$ and $\zeta$ are set by considering the signal-to-noise ratio (SNR) of the input image *I*(*x*, *y*, *z*) [18].

In the practical implementation, the 3-D VFC field $\mathbf {v}({x, y, z})=[u({x, y, z}), v({x, y, z}), w({x, y, z})]$ can be calculated by convolving the edge map *f*(*x*, *y*, *z*) with each component of the vector field kernel $\mathbf {k}({x, y, z})=[u_k({x, y, z}), v_k({x, y, z}), w_k({x, y, z})]$ [18]:

17 $$u({x, y, z})= f({x, y, z}) \otimes u_k({x, y, z}),$$

18 $$v({x, y, z}) = f({x, y, z}) \otimes v_k({x, y, z}),$$

19 $$w({x, y, z})= f({x, y, z}) \otimes w_k({x, y, z}).$$

The continuous vector field kernel $\mathbf {k}({x, y, z})$ is approximated by a discrete matrix defined on a 3-D grid [18]:

20 $$\{\mathbf {k}(x,y,z)~|x,y,z=-R,\ldots ,-1,0,1,\ldots ,R\},$$

where *R* is the chosen kernel radius.

To the best of our knowledge, all current work on deformable models employs VFC as force fields rather than potential energy. To tackle the main issues of the traditional external energy, we extend it for greedy algorithm as external potential energy. Specifically, we make the following enhancements to the original VFC method:

1. The magnitude of the VFC field $\left\| \mathbf {v}({x, y, z})\right\|$ is used as external energy instead of the force field itself.
2. One major drawback of the original VFC method is that the generated VFC fields are dominated by the strong edges, this will make the weak yet important edges smooth out along with the noise and cause leakage problem [18]. The GVF fields presented in [19] have successfully avoided this leakage problem and can retain the weak edges, as can be seen from Fig. 3(b) in [18]. It is mainly because in GVF, all the edges generated from different features are treated equally, which is achieved by normalizing the used edge maps to the range [0, 1] during GVF computations. After this normalization step, the weak edges will thus have similar magnitude to that of the strong edges, resulting in equal importance between them. Considering that no noise is presented in our binary input images, we propose also to normalize the edge map *f*(*x*, *y*, *z*) to [0, 1] before the computation of VFC energy, in order to alleviate the leakage problem and preserve the weak yet important edges.

Therefore, the VFC energy at vertex $P_i$ can be defined as:

21 $$\varvec{E}_{VFC}(P_i) = {\left\| \mathbf {v}(P_i)\right\| }.$$

When vertex $P_i$ is within the volumetric image, its external energy can be derived through a trilinear interpolation of the external energy at its neighboring image grid points; otherwise, to keep the deformable simplex meshes from moving out of the volumetric image, its VFC energy is set to be the maximal value of the external energy in the volumetric image [21]. When VFC is used as potential energy, we can pre-compute it and store its field magnitude as gray-level images, this can help greatly reduce the computation time of the VFC, while this does not hold when VFC is used as force fields.

### Statistical shape model construction

Using our proposed greedy algorithm-based deformable simplex meshes, we obtain a set of *K* corresponding femur training shapes $\{M_i~|~i=1,2,\ldots ,K\}$, and each shape $M_i$ is represented by a shape vector $\mathbf {x}_i$ with *N* landmark points. Firstly, all training shapes are spatially aligned into a common coordinate frame using the generalized Procrustes analysis (GPA) [29]. We define the corresponding covariance matrix as:

22 $$\mathbf {S} = \frac{1}{K-1} \sum _{i=1}^{K} (\mathbf {x}_i - \bar{\mathbf {x}})(\mathbf {x}_i -\bar{ \mathbf{x}})^T,$$

where $\bar{\mathbf {x}}$ is the mean shape vector of all subjects: $\bar{\mathbf {x}} = \frac{1}{K} { \sum _{i=1}^{K} { \mathbf {x}_i } }$. Then the principal component analysis (PCA)-based statistical shape model (SSM) can be built by an eigen-decomposition on the covariance matrix $\mathbf {S}$:

23 $$\mathbf {S} = \mathbf {U}\mathbf {D}\mathbf {U}^T,$$

where columns of matrix of $\mathbf {U}$ form the principal modes of variation $\phi _m$, and diagonal entries of $\mathbf {D}$ are their respective variances $\lambda _m$. Then any valid shapes of femur structure can be approximated by a linear combination of the first *c* modes of variation:

24 $$\mathbf { x } = \bar{\mathbf {x}} + \sum _{m=1}^{c} { b_m\phi _m },$$

where $c = \min \{ t ~ | ~ { \sum _{i=1}^{t} \lambda _t }/{ \sum _{i=1}^{K-1} \lambda _t } > 0.98 \}$, and $b_m$ is the shape parameter constrained to the interval $b_m \in \left[ -3\sqrt{\lambda _m}, 3\sqrt{\lambda _m}\right]$.

## Experimental setup

### Datasets and parameters

To evaluate the performance of our proposed deformable simplex meshes-based method for establishing shape correspondence, we applied it to construct the femur statistical shape models based on a femur database. The database consists of ten femur CT scans from 10 different patients (6 males, 4 females) with ground truth segmentation manually delineated by clinical experts. The ages of patients ranged from 30 to 71 years with an average age of 54 years. These CT scans were provided by the Weihai Municipal Hospital, and were acquired by use of a 64-row multidetector CT scanner (Brilliance 64; Philips Healthcare, Best, the Netherlands) with varied in-plane resolution between 0.58 and 0.67 mm, and a slice thickness of 1.5 mm.

In the experiments, the first magnitude function $m_1(\mathbf {x})$ is employed for the computation of VFC energy. The parameter settings of our proposed method are as follows: the weighting parameters in Eq. 8 $\alpha =0.4$, $\beta =1.0$, the width of the cubic window $w=11$; the vector field kernel radius $R = 256$, $\gamma =1.7$, and $\varepsilon = 10^{-8}$ for VFC energy. All these parameters are the same for all the training data. The parameters for the deformable model were chosen empirically according to that used in our previous paper [21], where we thoroughly evaluated the effect of these parameters on the final segmentation accuracy via the metric of mean radial error (MRE); while for VFC energy, the parameters were empirically set to the values suggested in the original VFC paper [18], where the authors experimentally showed the effect of these parameters on the final segmentation accuracy via the metric of root mean square error (RMSE).

### Compared method

We compared our proposed method with the classical spherical harmonics (SPHARM) shape correspondence method [8]. Although the SPHARM method is not being proposed recently, the reason we chose this method for comparison is that it is still quite efficient and popular, especially for the analysis of brain morphology structures in the neuroimaging community [30]. Moreover, the SPHARM method is still frequently cited by researchers for comparative experiments [30–32]. Since the SPHARM method requires the input shape to be of spherical topology (i.e., objects without holes), all holes in the training images must be filled firstly. The SPHARM method maps all the input shapes to a common parameter domain (i.e., the unit sphere) using an area-preserving spherical parameterization. It means that every vertex on the input surface is mapped to a unique point on the unit sphere using a spherical coordinate system. By using these spherical parameterizations, a unique shape descriptor coefficient can be derived for each input shape based on SPHARM basis functions. Point correspondence in the parameter domain is then determined by rotating the spherical parameterization, such that the poles of the input shape described by the first order coefficient (an ellipsoid) are coinciding with the poles of the parameterization. Finally the corresponded training shapes are derived by uniformly sampling the rotated spherical parameterization via icosahedron subdivision.

## Results

### Qualitative analysis

**Fig. 4:**
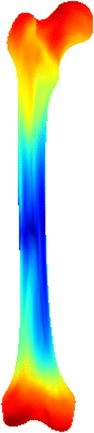
3D visualization of the shape variation on the surface for the constructed femur shape model. The variation spans from small (*blue*) to large (*red*)

**Fig. 5:**
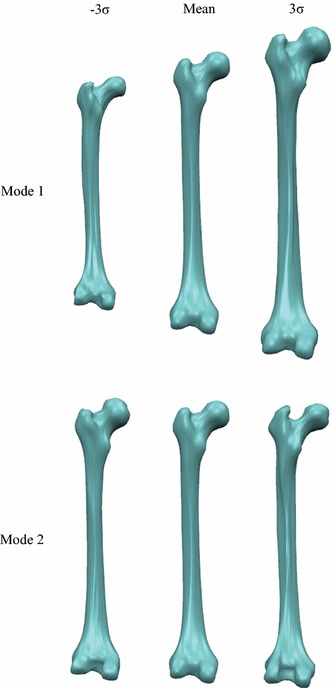
The first two principal modes of variation for the constructed femur shape model. Each row shows the variation of a specific mode between $-3\sigma$ and $+3\sigma$

Figure 4 shows 3D visualization of the shape variation on the surface for the constructed femur shape model. We can see that the upper extremity and lower extremity of the femur have more variation than other parts of the femur surface, while the body of the femur (middle parts) only has little variation (shown in dark blue color). Figure 5 illustrates the first two principal modes of variation for the constructed femur shape model. Each row shows the variation of a specific mode between $-3\sigma$ and $+3\sigma$. It can be seen that the first mode accounts for the whole volume size of the femur structure, while the second mode mainly represents the change of femoral neck and greater trochanter. These demonstrate that we have successfully constructed the femur shape model by using our proposed deformable simplex meshes-based method.

### Quantitative analysis

Compactness, generalization ability, and specificity are the three standard metrics for assessing the quality of a constructed shape model [33]. In this section, we use all these three metrics to quantitatively evaluate the quality of the constructed femur shape model by using our proposed method.

#### Compactness

A compact shape model should the one that has little variance and can accurately reconstruct new shape instances with few shape parameters. Thus, the compactness is defined as the cumulative variance of the *M*th mode used in the shape reconstruction [33]:

25 $$C(M) = \sum _{m=1}^{M} {\lambda _m},$$

where $\lambda _m$ is the *m*th eigenvalue. For the compactness, the smaller the value is, the better the constructed shape model.

**Fig. 6:**
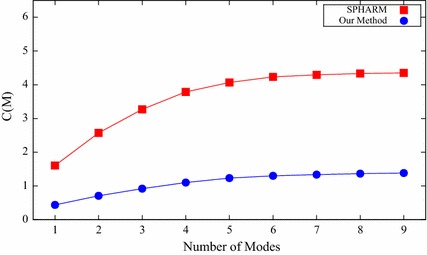
Compactness for both SPHARM and our proposed method

Figure 6 shows the compactness for both SPHARM and our proposed method with varying number of modes of variation. For all the employed number of modes, our method achieves better compactness than the SPHARM method. Table 1 shows the compactness for the two compared shape prior modeling methods with nine modes. Specifically, the compactness of our method is 1.38, and SPHARM method’s compactness is more than three times that of our method. Therefore, the shape model constructed by our method is more compact than that of the SPHARM method.

**Table 1:** The compactness for the two compared shape prior modeling methods with nine modes

Method	*C* (*M*)
SPHARM	4.35
*Our method*	1.38

#### Generalization ability

The generalization ability quantifies the ability of the constructed shape model to represent new shape instances of the same structure class. It is measured based on the training data by performing leave-one-out tests. Specifically, a shape model is built by using all but one training shape $\mathbf {x}_i$, and then the constructed model is employed to reconstruct the excluded shape $\mathbf {x}_i$. The approximation error is defined as the distance between the excluded shape $\mathbf {x}_i$ and its reconstructed shape $\mathbf {x}_i^{'}$. The generalization ability is the average approximation error of all the performed *K* tests [33]:

26 $$G(M) = \frac{1}{K} \sum _{i=1}^{K} { \Vert \mathbf {x}_i - \mathbf {x}_i^{'}(M) \Vert }^2,$$

where the reconstructed shape $\mathbf {x}_i^{'}(M)$ is defined as a linear combination of the first *M* modes of variation:

27 $$\mathbf {x}_i^{'}(M) = \bar{\mathbf {x}} + \sum _{m=1}^{M} { b_m\phi _m }.$$

And a smaller value of the generalization ability indicates a better constructed shape model.

**Fig. 7:**
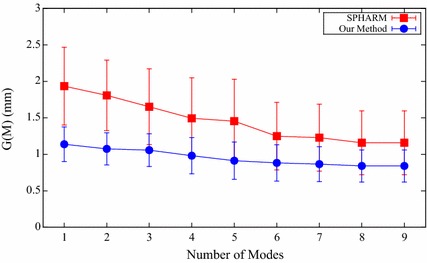
Generalization ability for both SPHARM and our proposed method

The generalization ability for both SPHARM and our proposed method is shown in Fig. 7 with different number of modes of variation. And our method shows consistently better generalization ability than the SPHARM method in all the used number of modes. The mean and standard deviation of the generalization ability for the two compared shape prior modeling methods are shown in Table 2 by using nine modes. The generalization ability of our method is 0.84 mm, while the generalization ability of SPHARM method is nearly 50% higher than that of our method. These demonstrate that our method can reconstruct new shape instances more accurately than the SPHARM method.

**Table 2:** The mean and standard deviation of the generalization ability for the two different shape prior modeling methods with nine modes

Method	*G* (*M*) (mm)
SPHARM	1.16 ± 0.44
*Our method*	0.84 ± 0.22

#### Specificity

The specificity measures the validity of shape instances generated by the constructed shape model. It is measured by generating a large set of *N* shape instances using the constructed model. The approximation error is defined as the distance between the generated shape instance $\mathbf {x}_j$ and its most similar sample in the training data $\mathbf {x}_j^{'}$. The specificity is the average approximation error of all the generated *N* shape instances [33]:

28 $$S(M) = \frac{1}{N} \sum _{j=1}^{N} { \Vert \mathbf {x}_j(M) - \mathbf {x}_j^{'} \Vert }^2,$$

where the shape instance $\mathbf {x}_j$ is generated by choosing random values for the first *M*’s shape parameter $\mathbf {b}$ from the range $b_m \in \left[ -3\sqrt{\lambda _m}, 3\sqrt{\lambda _m}\right]$:

29 $$\mathbf {x}_j(M) = \bar{\mathbf {x}} + \sum _{m=1}^{M} { b_m\phi _m },$$

and the most similar sample $\mathbf {x}_j^{'}$ is defined as:

30 $$\mathbf {x}_j^{'} = {\mathop{\text{arg min}}\limits_{k \in [1, K]}} \,{ \Vert \mathbf {x}_j(M) - \mathbf {x}_k \Vert }^2.$$

A value of *N* =  10,000 was used to obtain the results reported in this study. For the specificity, a smaller value means a better constructed shape model.

**Fig. 8:**
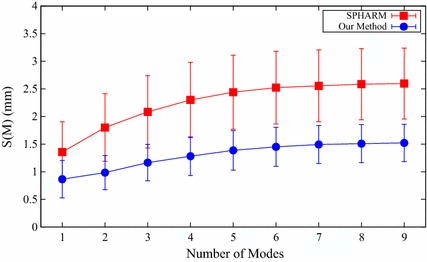
Specificity for both SPHARM and our proposed method

Figure 8 illustrates the specificity for both SPHARM and our proposed method with various number of modes of variation. In all the used number of modes, our method shows better specificity than the SPHARM method. Table 3 summarizes the mean and standard deviation of the specificity for the two compared shape prior modeling methods with nine modes. In particular, the specificity of our method is 1.52 mm, and SPHARM method’s specificity is more than 50% higher than that of our method. These indicate that the shape instances generated by the model of our method is more specific to the modeled structure than that of the SPHARM method.

**Table 3:** The mean and standard deviation of the specificity for the two different shape prior modeling methods with nine modes

Method	*S* (*M*) (mm)
SPHARM	2.60 ± 0.64
*Our method*	1.52 ± 0.34

Through all the above quantitative comparative results of the three employed metrics, we can conclude that the quality of the constructed femur shape models by using the proposed method is much better than that of the classical SPHARM method. Therefore, our method can be employed for effective femur shape model construction.

#### Computational time

The proposed method was implemented in C++^1^ and tested on a PC with an Intel processor and 2 GB of RAM. Our proposed method takes an average of about 15 min to establish shape correspondence for each shape. For comparison, the time taken by the SPHARM method for the same task is about 1 h in average. Thus our proposed method achieves much higher computational efficiency than the SPHARM method.

## Conclusion

In this paper, we have proposed a novel mesh-to-volume registration based method for automatically establishing shape correspondence. It integrates the computational simplicity of simplex meshes, speed of the greedy algorithm, and robustness of VFC in a unified system. Through extensive experiments on ten femur CT scans from ten different patients, we demonstrate that the quality of the constructed femur shape models by using the proposed method is much better than that of the classical SPHARM method. Moreover, the proposed method achieves much higher computational efficiency than the SPHARM method. Therefore, the proposed method can be employed for effective femur shape model construction.

For this study, we mainly focus on a new method for automatically establishing shape correspondence, rather than a direct extension of an existing method, thus few similar methods are available for direct comparisons. Nevertheless, we adapted the original implementation of the classical SPHARM method with fine-tuned parameter settings, and provided an extensive quantitative comparison based on the same femur CT datasets. Considering that no public femur CT datasets with ground truth are currently available, in our future work, we will implement more state-of-the-art methods and provide more comparative results. Furthermore, our future research will go in the direction of applying the constructed femur shape models in ASM based femur segmentation method as the shape prior models.

## Abbreviations

ASM: active shape model
SSM: statistical shape models
PCA: principal component analysis
VFC: vector field convolution
GVF: gradient vector flow
GMM: Gaussian mixture model
SPHARM: spherical harmonics
GPA: generalized Procrustes analysis
AFDM: adaptive-focus deformable model

## Footnotes

1 The source code is available at https://github.com/ivanshih/GA-DSM.
